# A randomized, placebo‐controlled trial evaluating the safety of excessive administration of kaempferol aglycone

**DOI:** 10.1002/fsn3.3499

**Published:** 2023-06-12

**Authors:** Minoru Akiyama, Tsubasa Mizokami, Hikaru Ito, Yasutaka Ikeda

**Affiliations:** ^1^ Saga Nutraceuticals Research Institute Otsuka Pharmaceutical Co., Ltd. Kanzaki‐gun Japan; ^2^ Otsu Nutraceuticals Research Institute Otsuka Pharmaceutical Co., Ltd. Otsu Japan

**Keywords:** excessive administration, horseradish (*Armoracia rusticana*) leaves, kaempferol, safety information

## Abstract

Kaempferol (KMP) is an important flavonoid in many fruits and vegetables. Preclinical studies on KMP have reported its pharmacological effects, including antimicrobial, antioxidant, anti‐inflammatory, antitumor, antidiabetic, myocardial protective, and neuroprotective effects. Additionally, some epidemiological studies have revealed a negative association between the consumption of KMP‐containing foods and the risk of developing several disorders, such as cancer and cardiovascular diseases. Thus, although a large body of literature has demonstrated the benefits of KMP supplementation, there are no reports of clinical trials evaluating the safety of KMP aglycone administration or KMP aglycone‐rich food consumption. The purpose of this study was to evaluate the safety of a high dose of KMP aglycone by administrating KMP aglycone‐containing supplements to healthy adults. This study had a randomized, double‐blind, placebo‐controlled design and a 4‐week duration. Participants were randomly allocated to the KMP (*n* = 24) or placebo (*n* = 24) group. For 4 weeks, the KMP group received a capsule containing 50‐mg KMP daily, a dose approximately five times higher than the estimated human dietary intake. The placebo group received a capsule containing cornstarch‐based powder daily. The general toxicity parameters were evaluated by examining the characteristics of the participants, hematological and blood biochemical parameters, general urinalysis, qualitative urine tests, and adverse events. No clinical changes were observed in anthropometric and blood pressure measurements or blood and urine parameters in the KMP group compared to those in the placebo group. Furthermore, no adverse events owing to KMP aglycone administration occurred. The study results revealed that the consumption of 50‐mg KMP aglycone daily for 4 weeks is safe in healthy adults.

## Graphical abstract

A kaempferol aglycone‐based supplement was developed using horseradish leaves. This is the first clinical evaluation of the safety of kaempferol aglycone intake. A 4‐week continuous intake of 50‐mg kaempferol aglycone/day was safe.

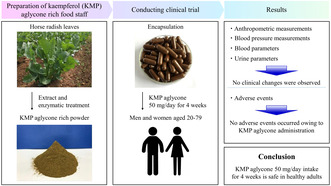

## 1 INTRODUCTION

Many dietary phytochemicals and their derivatives present various physiological functions. Among these compounds, flavonoids have garnered increasing attention owing to their antioxidant, cardioprotective, and immunomodulatory properties (Ruiz‐Iglesias et al., 2021). Consequently, their consumption as dietary supplements has increased dramatically (Skibola & Smith, 2000). While previous studies have focused on the immunomodulatory, vasodilatory, and anti‐inflammatory effects of flavonoids (Al‐Dashti et al., 2018; Ruiz‐Iglesias et al., 2021), recent flavonoid‐related research has explored their use in improving exercise performance (Al‐Dashti et al., 2018; Decroix et al., 2018). However, only a few studies have investigated their effects on adenosine triphosphate (ATP), the energy source that drives cellular processes, including muscle contraction, nerve impulse propagation, and chemical synthesis (Dunn & Grider, 2022). A decrease in performance during exercise is associated with a decrease in ATP content in the body (Bartlett et al., 2020), which is caused by a metabolic shift from aerobic to anaerobic respiration mediated by hypoxia‐inducible factor‐1α (HIF‐1α). Given that HIF‐1α is stabilized by hypoxia in the body (Kierans & Taylor, 2021), an increase in ATP content in the body by promoting aerobic respiration, an efficient ATP‐producing system, would improve exercise performance. Additionally, a decrease in ATP content in the body has been recently observed in various disorders (Boyman et al., 2020), suggesting that its increase could improve these diseases. Even in healthy people, aging is associated with decreased ATP content in the body (Ma & Li, 2015). Therefore, identifying methods to increase ATP content in the body is gaining attention.

In a previous study, kaempferol (KMP), a flavonol widely found in nature, was shown to enhance hypoxia‐induced HIF‐1α degradation and increase mitochondrial complex IV activity, thereby stimulating aerobic respiration and increasing ATP content in vitro and in vivo (Akiyama et al., 2022). However, clinical trials are required to clarify whether KMP increases ATP content and improves exercise performance or suppresses disease‐related malfunctions and age‐related decline in humans.

The safety and toxic profile of KMP aglycone has been evaluated in vitro and in vivo (Francis et al., 1989; Kimoto et al., 2022; MacGregor & Jurd, 1978; Niering et al., 2005; Yangzom et al., 2022). However, in human research, although there are reports of studies using KMP glycosides and conjugates, which are abundant in nature, to our knowledge, there are no reports of clinical trials evaluating the safety of KMP aglycone. Given that KMP aglycone is generally more absorbable than glycosides and conjugates (Imran et al., 2019) and considered useful as a functional ingredient, safety evaluation in clinical trials using aglycone or aglycone‐rich foods is necessary to validate the effects of KMP aglycone in humans. Therefore, in this study, a KMP aglycone supplement was developed in‐house, and a 4‐week, randomized, double‐blind, placebo‐controlled trial was conducted. The characteristics of the participants, hematological and blood biochemical parameters, general urinalysis, qualitative urine test, and adverse events were evaluated to assess the safety of a daily intake of 50‐mg KMP aglycone in healthy adults. This dosage is approximately five times higher than the estimated human dietary intake of KMP (Calderón‐Montaño et al., 2011). This study is the first clinical trial to evaluate the safety of a high‐dose KMP aglycone intake.

## 2 MATERIALS AND METHODS

### 2.1 Study design and participants

**FIGURE 1:**
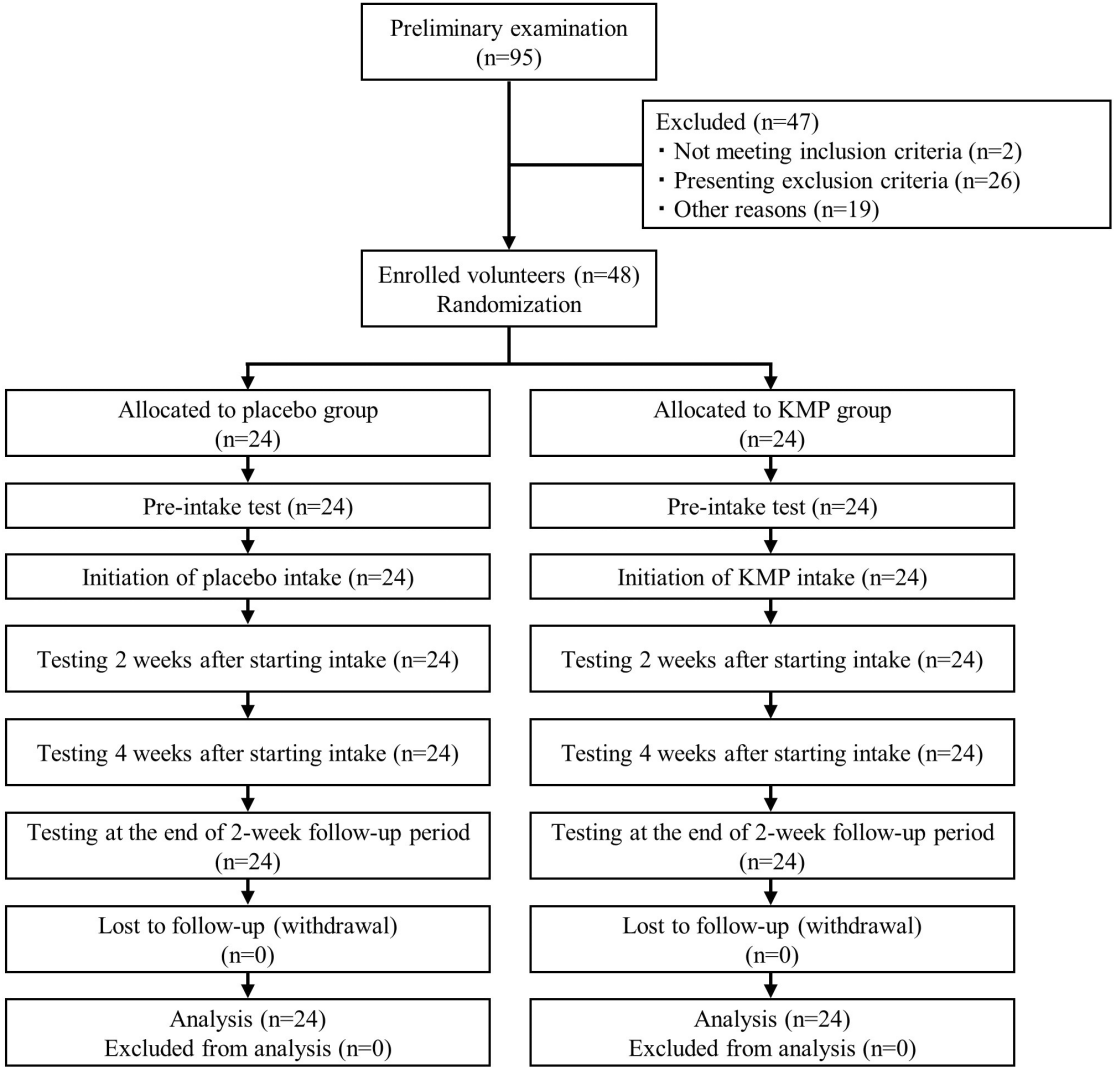
Consolidated standards of reporting trial diagram: enrollment, random assignment, and follow‐up of volunteers. KMP, kaempferol.

This randomized, double‐blind, placebo‐controlled clinical trial was preceded by a 2‐day preliminary examination period, where volunteers underwent physical examination, and their urinary KMP excretion rate was assessed. In total, 95 volunteers underwent preliminary examinations for enrolment. However, 48 healthy male and female volunteers were included in this placebo‐controlled, parallel study with a 4‐week intake and 2‐week follow‐up period (Figure 1). Participant eligibility was determined using the following inclusion criteria: men and women aged between 20 and 79 years at the time of consent for participation and individuals who were properly informed about the purpose and protocol of the study and could consent voluntarily, in writing, to participate, fully understanding its content. The exclusion criteria included past or current conditions affecting major organs; current drug treatment for a certain disease; food allergies; pregnancy, breastfeeding, or the possibility of being pregnant; and being considered unsuitable for participation by the principal investigator. Participants were instructed to continue their previous eating habits for the study duration. Another essential aspect of the study was the inclusion of a population with a wide age range, which enabled the proper assertation of the safety of the tested compound. The study population was stratified by age, with eight male and eight female participants in each of three brackets (20–39, 40–59, and 60–79), thus ensuring an equal sex ratio across the age groups. All participants provided written informed consent for participation. This study was approved by the ethics committee of AMC Nishi‐Umeda Clinic and was conducted according to the principles of the Declaration of Helsinki (approval number: RD2020‐01). Furthermore, the study protocol was registered with the University Hospital Medical Information Network Clinical Trial Registry (UMIN‐CTR; registration number: UMIN000041986).

### 2.2 Test products

The test powder, from enzyme‐treated horseradish (*Armoracia rusticana*) leaves, containing KMP aglycone was obtained from Otsuka Pharmaceutical Co., Ltd. (Tokyo, Japan) and comprised approximately 16.1% KMP aglycone as an active ingredient (Ikeda et al., 2020). It was formulated as a capsule filled with 25‐mg KMP aglycone. Indistinguishable placebo capsules were filled with cornstarch‐based powder.

### 2.3 Physical examinations and urinary KMP excretion rate

The physical examination items included height, weight, body mass index (BMI), blood pressure (systolic/diastolic), and pulse rate. The height was measured only during the preliminary examination period, and the BMI was calculated based on this measurement. During the preliminary examination period, participants ingested a single dose of 25‐mg KMP, and urine was collected up to 24 h after supplementation to assess KMP excretion levels.

### 2.4 Urine sampling

Urine samples were collected for up to 24 h after a single dose of 25‐mg KMP supplementation and stored at 4°C. All samples collected for each participant were pooled at the end of the 24‐h period. The volume of each sample was recorded, and samples were stored at −80°C until further analysis.

### 2.5 Extraction of KMP from urine

The urine samples (100 μL) were mixed with 100 μL of β‐glucuronidase solution, diluted in 0.2 M sodium acetate buffer (pH 5.0) for a final concentration of 50 units of β‐glucuronidase. After incubating the mixtures at 37°C for 0.5 h, 200 μL of 4% phosphate buffer containing apigenin‐d5 (internal standard; Toronto Research Chemicals) was added. Then, the urine samples were transferred to a conditioned 96‐well MCX‐Elute plate (Waters Corporation). After the samples were drawn through the sorbent bed, they were washed with 200 μL of 2% formic acid in water, followed by 200 μL of 40% methanol. KMP and the internal standard were eluted with 150 μL of methanol/acetonitrile (1:1) in another 96‐well elution plate for the final analysis.

### 2.6 Liquid chromatography‐mass spectrometry analysis of KMP in urine

Chromatographic separation of the injected 20‐μL sample was conducted using a reverse‐phased C18 analytical column (50 mm × 2 mm, 3‐μm particle size, Cadenza CD‐C18, Imtakt Co.), and liquid chromatographic separation was achieved using a high‐performance liquid chromatography (HPLC) system (Shiseido Nanospace). The mobile phase used was a mixture of 0.1% formic acid with water and acetonitrile, operated at a flow rate of 0.35 mL/min. The initial mobile phase composition (20% acetonitrile) was constant for 0.5 min, followed by a linear gradient to 95% acetonitrile for 1.5 min, maintained until 3.5 min, then changed to 20% acetonitrile at 3.6 min, and maintained until 5.5 min. Next, the samples separated using HPLC were analyzed using a Sciex API‐3000 tandem mass spectrometer equipped with an electrospray interface (Sciex).

### 2.7 Randomization and blinding

The gender, age, BMI, and urinary KMP excretion rate measured at the preliminary examination were used as stratification factors. The patients were randomly assigned to the test or placebo group using the stratified block randomization method. There were no significant differences between the two groups regarding the measured parameters. The test group received KMP supplement capsules (50‐mg KMP aglycon/two capsules/day), whereas the placebo group received placebo capsules (cornstarch‐based powder/two capsules/day). The investigator ensured that the two capsule types were indistinguishable. The relevant identification codes were printed on the pack containing each substance, then printed and sealed in an envelope, and delivered to the study outsource provider. The identification codes were sealed in an envelope and sent to the allocator, who confirmed that neither of the capsules could be identified by their appearance and/or smell. Next, the allocator replaced the KMP supplement and placebo codes with another control code that could not be easily identifiable, thereby blinding the study. The products were transferred to the test product manager. The control codes were tightly sealed in an envelope together with the correspondence table and kept by the allocator until the study was unblinded.

### 2.8 Blood and urine analysis

Hematology tests included white blood cell count (WBC), red blood cell count (RBC), hemoglobin (Hb), hematocrit (Ht), mean corpuscular volume (MCV), mean corpuscular hemoglobin (MCH), mean corpuscular hemoglobin concentration (MCHC), and platelet count (PLT). Biochemical examination of blood comprised the measurement of the values of total protein, albumin, total bilirubin, direct bilirubin, alkaline phosphatase (ALP), γ‐glutamyltranspeptidase (γ‐GTP), aspartate aminotransferase (AST), alanine aminotransferase (ALT), lactate dehydrogenase (LDH), creatine kinase (CK), C‐reactive protein (CRP), total cholesterol, high‐density lipoprotein cholesterol (HDL‐cho), low‐density lipoprotein cholesterol (LDL‐cho), triglyceride (TG), blood urea nitrogen (BUN), creatinine, uric acid (UA), sodium, potassium, chloride, calcium, phosphorus, glucose, and glycated hemoglobin (HbA_1c_). For general urinalysis, pH and specific gravity were measured. For qualitative urine tests, protein, glucose, urobilinogen, bilirubin, ketone body, and occult blood presence were measured. These assessments were performed at the beginning of the study, 2 and 4 weeks after the intake initiation, and during the follow‐up observational period.

### 2.9 Side effects and adverse events

All participants were monitored throughout the study for side effects and adverse events. Safety monitoring included a questionnaire on general health and the occurrence of any health‐related events. The physician evaluated the results of the interviews and diaries of participants at weeks 0, 2, 4, and at follow‐up and determined the occurrence of a possible relationship between any observed adverse events and KMP/placebo intake while remaining blinded to group allocation.

### 2.10 Statistical analysis

Values analyzed in this study are expressed as mean ± standard deviation. For within‐group comparison, continuous variables were analyzed using a linear mixed‐effects model with treatment and time of determination as fixed factors and participants as random factors. Considering the mixed model of repeated measures approach, missing data were not inputted in the primary model. For categorical data, statistically significant differences between study groups were examined using Fisher's exact test. All statistical analyses were performed using SAS software version 9.4 (SAS Institute), and statistical significance was set at *p* values < .05.

## 3 RESULTS

### 3.1 Participants

Forty‐eight participants were enrolled in this trial, and all participants completed the trial (Figure 1). The baseline characteristics of the participants are shown in Table 1. No significant baseline differences were observed between the groups for any measured parameter.

**TABLE 1:** Characteristics of participants.

Variable	Placebo group			KMP group		
	Overall	Men	Women	Overall	Men	Women
*N*	24	12	12	24	12	12
Age (years)	48.5 ± 15.1	48.7 ± 15.5	48.4 ± 15.4	49.5 ± 16.1	50.8 ± 16.0	48.3 ± 16.9
Height (cm)	163.6 ± 9.4	169.6 ± 7.1	157.7 ± 7.5	164.2 ± 8.3	169.6 ± 7.0	158.7 ± 5.5
Weight (kg)	58.1 ± 8.6	64.8 ± 6.3	51.4 ± 4.0	58.4 ± 9.5	64.5 ± 7.6	52.3 ± 7.2
BMI (kg/m^2^)	21.6 ± 1.8	22.5 ± 1.5	20.8 ± 1.6	21.5 ± 2.1	22.3 ± 1.6	20.7 ± 2.3
Urinary KMP excretion rate (%)	9.4 ± 4.6	9.2 ± 4.1	9.6 ± 5.3	9.3 ± 4.7	9.3 ± 4.2	9.3 ± 5.3

*Note*: Data are presented as mean ± standard deviation.

Abbreviations: BMI, body mass index; KMP, kaempferol.

### 3.2 Physical examinations

Table 2 shows the baseline physical characteristics of the participants, measured at week 0, and the changes in the investigated parameters throughout the study. At follow‐up, systolic blood pressure, diastolic blood pressure, and pulse in the placebo group were significantly increased compared with those at baseline, and systolic blood pressure was significantly higher in the placebo group than in the KMP group. No other significant differences were observed between the groups at any point. Neither group had significant changes compared with baseline for weight, BMI, body fat percentage, and lean body mass.

**TABLE 2:** Changes in the characteristics of participants.

Item	Unit	Group	Weeks			Follow‐up period
			0	2	4	
Weight	kg	Placebo	58.3 ± 8.8	58.3 ± 8.6	58.5 ± 8.7	58.3 ± 8.8
		KMP	58.6 ± 9.6	58.4 ± 9.4	58.5 ± 9.6	58.3 ± 9.2
BMI	kg/m^2^	Placebo	21.7 ± 1.9	21.7 ± 1.8	21.8 ± 1.8	21.7 ± 1.9
		KMP	21.6 ± 2.2	21.6 ± 2.1	21.6 ± 2.2	21.5 ± 2.1
Percent of body fat	%	Placebo	26.0 ± 5.6	26.0 ± 5.7	26.1 ± 5.7	26.0 ± 5.7
		KMP	25.8 ± 5.6	25.7 ± 5.7	25.7 ± 5.8	25.7 ± 5.8
Lean body mass	kg	Placebo	43.4 ± 8.4	43.4 ± 8.4	43.4 ± 8.4	43.3 ± 8.4
		KMP	43.5 ± 8.2	43.5 ± 8.1	43.5 ± 8.1	43.4 ± 8.0
Systolic blood pressure	mmHg	Placebo	113.4 ± 11.2	114.6 ± 10.4	112.4 ± 10.0	119.5 ± 9.9*
		KMP	112.8 ± 13.4	112.0 ± 12.0	111.8 ± 14.0	114.2 ± 13.1^#^
Diastolic blood pressure	mmHg	Placebo	67.5 ± 6.3	66.3 ± 7.9	68.5 ± 8.2	71.5 ± 6.3*
		KMP	67.0 ± 9.2	66.6 ± 9.1	68.4 ± 7.9	68.3 ± 8.1
Pulse	bpm	Placebo	67.7 ± 9.2	69.6 ± 9.2	68.9 ± 10.2	70.9 ± 11.1*
		KMP	69.4 ± 8.6	71.1 ± 8.7	70.5 ± 10.6	71.4 ± 10.5

*Note*: Data are presented as mean ± standard deviation. *p < .05 versus week 0, ^#^ *p* < .05 versus placebo, mixed model for repeated measures method.

Abbreviations: BMI, body mass index; bpm, beats per minute; KMP, kaempferol.

### 3.3 Hematology tests

No significant differences were observed between groups for WBC and RBC values throughout the study period (Table 3). In the placebo group, compared with baseline values, significant changes were observed in Ht values at week 4, in Hb, MCH, and MCHC values at follow‐up, and in MCV values at weeks 2, 4, and follow‐up. In the KMP group, compared with baseline values, significant changes were observed in Ht and MCHC values at weeks 2 and 4, in PLT values at week 2 and follow‐up, and in MCV values at weeks 2, 4, and follow‐up. In addition, MCHC in the KMP group was significantly higher than that in the placebo group at weeks 0 and 4. However, a physician confirmed that all changes were not clinically relevant.

**TABLE 3:** Changes in hematological parameters.

Item	Unit	Group	Weeks			Follow‐up period
			0	2	4	
WBC	/μL	Placebo	5192 ± 1348	5513 ± 1583	5338 ± 1126	5138 ± 1194
		KMP	5108 ± 1463	5083 ± 1299	5275 ± 1625	4967 ± 1479
RBC	× 10^4^/μL	Placebo	451 ± 38	454 ± 35	455 ± 38	455 ± 37
		KMP	438 ± 36	445 ± 36	443 ± 37	445 ± 32
Hb	g/dL	Placebo	13.7 ± 1.1	13.8 ± 1.1	13.9 ± 1.2	14.0 ± 1.1*
		KMP	13.6 ± 1.2	13.7 ± 1.2	13.8 ± 1.2	13.8 ± 1.0
Ht	%	Placebo	42.2 ± 3.3	42.7 ± 3.1	43.2 ± 3.1*	42.0 ± 3.1
		KMP	41.4 ± 3.3	42.9 ± 3.4*	42.6 ± 3.4*	41.7 ± 2.9
MCV	fL	Placebo	93.7 ± 4.5	94.3 ± 4.9*	95.0 ± 5.0*	92.4 ± 4.7*
		KMP	94.5 ± 3.6	96.5 ± 3.4*	96.1 ± 4.2*	93.8 ± 3.4*
MCH	pg	Placebo	30.4 ± 1.6	30.6 ± 1.6	30.6 ± 1.6	30.7 ± 1.6*
		KMP	31.1 ± 1.2	30.9 ± 1.3	31.2 ± 1.5	31.0 ± 1.1
MCHC	%	Placebo	32.4 ± 0.8	32.4 ± 0.9	32.2 ± 0.8	33.2 ± 0.8*
		KMP	32.9 ± 0.8^#^	32.1 ± 0.8*	32.5 ± 0.8*^,#^	33.1 ± 0.7
PLT	× 10^4^/μL	Placebo	24.5 ± 4.7	24.4 ± 4.9	23.6 ± 5.3	24.9 ± 4.9
		KMP	23.7 ± 4.4	25.0 ± 5.0*	24.7 ± 4.0	25.1 ± 5.0*

*Note*: Data are presented as mean ± standard deviation. *p < .05 versus week 0, ^#^ *p* < .05 versus placebo, mixed model for repeated measures method.

Abbreviations: Hb, hemoglobin; Ht, hematocrit; KMP, kaempferol; MCH, mean corpuscular hemoglobin; MCHC, mean corpuscular hemoglobin concentration; MCV, mean corpuscular volume; PLT, platelet count; RBC, red blood cell count; WBC, white blood cell count.

### 3.4 Biochemical examination of blood

The results of blood biochemistry tests are shown in Table 4. In the placebo group, compared with baseline values, significant changes were observed in ALP, AST, ALT, and P values at week 4, in Na and HbA_1c_ values at follow‐up, in Cl values at weeks 2 and 4, and in K values at week 2 and follow‐up. In the KMP group, compared with baseline values, significant changes were observed in total protein, HDL‐cho, LDL‐cho, and UA values at week 4, in CK, Na, K, Ca, and glucose values at follow‐up, and in HbA_1c_ values at week 4 and follow‐up. Total bilirubin, ALP, and K values at follow‐up and direct bilirubin values at week 2 and follow‐up were significantly higher in the KMP than in the placebo group. Conversely, Na at week 2, AST and ALT at week 4, P at weeks 2 and 4, and γ‐GTP values at week 4 and follow‐up were significantly lower in the KMP group than in the placebo group. The physician confirmed that all changes were not clinically relevant.

**TABLE 4:** Changes in blood biochemical parameters.

Item	Unit	Group	Weeks			Follow‐up period
			0	2	4	
Total protein	g/dL	Placebo	7.1 ± 0.3	7.0 ± 0.3	7.2 ± 0.4	7.2 ± 0.3
		KMP	7.1 ± 0.4	7.2 ± 0.4	7.2 ± 0.3*	7.1 ± 0.3
Albumin	mg/dL	Placebo	4.4 ± 0.2	4.4 ± 0.2	4.5 ± 0.3	4.5 ± 0.3
		KMP	4.4 ± 0.2	4.4 ± 0.3	4.5 ± 0.3	4.5 ± 0.3
Total bilirubin	mg/dL	Placebo	0.80 ± 0.41	0.74 ± 0.33	0.82 ± 0.37	0.76 ± 0.31
		KMP	0.75 ± 0.31	0.72 ± 0.24	0.80 ± 0.27	0.81 ± 0.29^#^
Direct bilirubin	mg/dL	Placebo	0.22 ± 0.11	0.21 ± 0.09	0.23 ± 0.09	0.21 ± 0.09
		KMP	0.21 ± 0.09	0.22 ± 0.07^#^	0.23 ± 0.08	0.23 ± 0.08^#^
ALP	U/L	Placebo	177.2 ± 41.1	178.2 ± 40.8	188.0 ± 46.2*	183.6 ± 41.5
		KMP	201.2 ± 76.5	196.9 ± 67.8	209.7 ± 79.7	198.5 ± 80.3^#^
γ‐GTP	U/L	Placebo	21.4 ± 12.5	21.5 ± 13.1	22.6 ± 14.1	22.8 ± 16.8
		KMP	20.9 ± 10.6	21.1 ± 10.3	20.0 ± 9.8^#^	19.7 ± 9.0^#^
AST	U/L	Placebo	20.5 ± 5.2	20.8 ± 4.5	21.9 ± 4.3*	21.1 ± 4.2
		KMP	20.7 ± 5.3	20.5 ± 4.9	20.4 ± 5.7^#^	20.8 ± 5.9
ALT	U/L	Placebo	18.3 ± 8.5	19.3 ± 9.8	21.1 ± 9.5*	19.5 ± 10.7
		KMP	19.5 ± 11.4	20.3 ± 13.6	18.8 ± 12.1^#^	18.9 ± 12.9
LDH	U/L	Placebo	167.3 ± 28.2	168.9 ± 31.3	167.1 ± 29.3	164.5 ± 27.9
		KMP	162.8 ± 29.1	161.5 ± 28.7	164.0 ± 29.7	161.8 ± 30.9
CK	U/L	Placebo	96.7 ± 46.2	101.7 ± 56.9	98.6 ± 41.1	99.3 ± 41.9
		KMP	94.4 ± 41.8	93.1 ± 43.3	96.3 ± 43.1	105.4 ± 52.9*
CRP	mg/dL	Placebo	0.06 ± 0.11	0.04 ± 0.03	0.10 ± 0.25	0.06 ± 0.09
		KMP	0.08 ± 0.12	0.12 ± 0.26	0.17 ± 0.60	0.07 ± 0.14
Total cholesterol	mg/dL	Placebo	190.7 ± 27.2	190.4 ± 27.9	193.5 ± 27.9	191.5 ± 25.1
		KMP	192.1 ± 23.9	194.5 ± 20.4	197.7 ± 26.4	196.3 ± 29.1
HDL‐cho	mg/dL	Placebo	70.6 ± 12.3	69.5 ± 10.0	71.5 ± 11.6	69.6 ± 11.9
		KMP	67.8 ± 12.5	67.6 ± 13.3	70.7 ± 15.2*	68.7 ± 13.8
LDL‐cho	mg/dL	Placebo	103.2 ± 21.5	103.5 ± 24.3	105.1 ± 23.4	103.3 ± 20.9
		KMP	106.7 ± 19.7	109.8 ± 22.8	110.7 ± 24.4*	109.5 ± 23.5
TG	mg/dL	Placebo	60.6 ± 28.1	68.9 ± 37.4	72.1 ± 35.6	65.6 ± 39.5
		KMP	72.2 ± 36.4	70.4 ± 26.0	69.7 ± 42.4	62.3 ± 23.7
BUN	mg/dL	Placebo	13.5 ± 2.6	14.0 ± 2.5	13.7 ± 2.9	13.6 ± 2.8
		KMP	13.6 ± 3.9	13.6 ± 3.6	13.1 ± 3.4	13.0 ± 3.4
Creatinine	mg/dL	Placebo	0.77 ± 0.15	0.78 ± 0.15	0.76 ± 0.15	0.76 ± 0.15
		KMP	0.76 ± 0.12	0.78 ± 0.13	0.75 ± 0.13	0.76 ± 0.11
UA	mg/dL	Placebo	4.8 ± 1.0	4.8 ± 1.0	4.8 ± 1.1	4.7 ± 1.0
		KMP	4.9 ± 1.2	4.7 ± 1.1	4.7 ± 1.0*	4.7 ± 0.9
Na	mEq/L	Placebo	140.6 ± 1.3	141.0 ± 1.5	140.5 ± 1.4	139.7 ± 1.4*
		KMP	140.3 ± 1.9	140.3 ± 1.8^#^	139.9 ± 1.7	139.3 ± 2.0*
K	mEq/L	Placebo	4.2 ± 0.4	4.0 ± 0.3*	4.0 ± 0.3	4.4 ± 0.4*
		KMP	4.2 ± 0.3	4.1 ± 0.3	4.1 ± 0.4	4.6 ± 0.5*^,#^
Cl	mEq/L	Placebo	103.3 ± 1.9	102.6 ± 1.7*	102.1 ± 1.6*	102.9 ± 1.7
		KMP	102.5 ± 2.1	102.3 ± 2.0	102.0 ± 1.9	102.6 ± 2.0
Ca	mg/dL	Placebo	9.2 ± 0.3	9.2 ± 0.2	9.2 ± 0.3	9.2 ± 0.3
		KMP	9.4 ± 0.3	9.3 ± 0.3	9.3 ± 0.3	9.2 ± 0.3*
P	mg/dL	Placebo	3.1 ± 0.6	3.3 ± 0.6	3.4 ± 0.5*	3.2 ± 0.4
		KMP	3.0 ± 0.5	3.0 ± 0.8^#^	3.2 ± 0.6^#^	3.1 ± 0.5
Glucose	mg/dL	Placebo	88.8 ± 7.6	87.5 ± 7.6	86.4 ± 10.1	89.3 ± 10.5
		KMP	91.5 ± 8.4	90.1 ± 9.6	89.3 ± 8.7	88.2 ± 9.1*
HbA_1c_	%	Placebo	5.2 ± 0.3	5.2 ± 0.3	5.2 ± 0.2	5.3 ± 0.2*
		KMP	5.3 ± 0.3	5.3 ± 0.3	5.3 ± 0.3*	5.4 ± 0.3*

*Note*: Data are presented as mean ± standard deviation. *p < .05 versus week 0, ^#^ *p* < .05 versus placebo, mixed model for repeated measures method.

Abbreviations: ALP, alkaline phosphatase; ALT, alanine aminotransferase; AST, aspartate transaminase; BUN, blood urea nitrogen; CK, creatine kinase; CRP, C‐reactive protein; HbA_1c_, glycated hemoglobin A_1c_; HDL‐cho, high‐density lipoprotein cholesterol; KMP, kaempferol; LDH, lactate dehydrogenase; LDL‐cho, low‐density lipoprotein cholesterol; TG, triglyceride; UA, uric acid; γ‐GTP, γ‐glutamyl transferase.

### 3.5 General urinalysis and qualitative urine test

Urinary pH values significantly increased in the placebo group at follow‐up than at baseline. Urinary pH was significantly higher in the KMP group than in the placebo group at week 2 (Table 5). The qualitative urine analysis revealed hematuria in the placebo group at weeks 0 and 4 and proteinuria and ketonuria in the KMP group at week 2. However, there were no significant differences between the groups regarding the frequency of these adverse reactions (Table 6).

**TABLE 5:** Changes in general urinalysis.

Item	Group	Weeks			Follow‐up period
		0	2	4	
pH	Placebo	5.5 ± 0.6	5.5 ± 0.5	5.6 ± 0.6	5.9 ± 0.6*
	KMP	5.9 ± 0.7	6.0 ± 0.8^#^	5.9 ± 0.6	6.1 ± 0.8
Specific gravity	Placebo	1.016 ± 0.009	1.016 ± 0.009	1.015 ± 0.007	1.018 ± 0.009
	KMP	1.013 ± 0.007	1.014 ± 0.007	1.015 ± 0.008	1.015 ± 0.007

*Note*: Data are presented as mean ± standard deviation. *p < .05 versus week 0, ^#^ *p* < .05 versus placebo, mixed model for repeated measures method.

Abbreviation: KMP, kaempferol.

**TABLE 6:** Changes in qualitative urine test.

Item	Group	Weeks						Follow‐up period	
		0		2		4			
		Within	Without	Within	Without	Within	Without	Within	Without
Protein	Placebo	24	0	24	0	24	0	24	0
	KMP	24	0	23	1	24	0	24	0
Glucose	Placebo	24	0	24	0	24	0	24	0
	KMP	24	0	24	0	24	0	24	0
Urobilinogen	Placebo	24	0	24	0	24	0	24	0
	KMP	24	0	24	0	24	0	24	0
Bilirubin	Placebo	24	0	24	0	24	0	24	0
	KMP	24	0	24	0	24	0	24	0
Ketone body	Placebo	24	0	24	0	24	0	24	0
	KMP	24	0	23	1	24	0	24	0
Occult blood reaction	Placebo	23	1	24	0	23	1	24	0
	KMP	24	0	24	0	24	0	24	0

*Note*: Data are presented as the number of participants.

Abbreviation: KMP, kaempferol.

### 3.6 Side effects and adverse events

No side effects were attributable to the test powder in any participant. Adverse events occurred in both groups: placebo group (*n* = 4, 6 events; abdominal pain, dry eyes, stomatitis, headache, and cough); KMP group (*n* = 6, 10 events; premenstrual syndrome [abdominal pain, malaise, sleepiness], headache, red eye, stomach pain, malaise, sore throat, common cold [sore throat, cough], right foot sprain, lower back pain, and pain in the left leg) (Table 7).

**TABLE 7:** List of adverse events.

Group	Subject no.	Symptoms and findings	Grade	Seriousness	Causal relationship
Placebo	2	Abdominal pain	Mild	Not serious	Not related
	17	Dry eyes	Moderate	Not serious	Not related
	17	Stomatitis	Moderate	Not serious	Not related
	46	Abdominal pain	Mild	Not serious	Not related
	47	Headache	Mild	Not serious	Not related
	47	Cough	Mild	Not serious	Not related
KMP	4	Premenstrual syndrome (abdominal pain, malaise, sleepiness)	Mild	Not serious	Not related
	4	Headache	Mild	Not serious	Not related
	15	Red eye	Moderate	Not serious	Not related
	22	Stomach pain	Mild	Not serious	Not related
	28	Malaise	Mild	Not serious	Not related
	31	Sore throat	Mild	Not serious	Not related
	31	Common cold (sore throat, cough)	Mild	Not serious	Not related
	40	Right foot sprain	Moderate	Not serious	Not related
	40	Lower back pain	Moderate	Not serious	Not related
	40	Pain in the left leg	Moderate	Not serious	Not related

Abbreviation: KMP, kaempferol.

## 4 DISCUSSION

Although growing evidence supports the potential use of KMP for the prevention and/or treatment of several diseases, to our knowledge, no clinical trials have been conducted to verify these effects using KMP aglycone or KMP aglycone‐rich foods. This may be because KMP exists in nature mainly as glycosides and conjugates, and no food or food material contains a large amount of aglycone. Consequently, there are no clinical data on the safety of KMP intake. In vitro studies have reported the possibility of pro‐oxidant effects (autoxidation, pro‐oxidation) with high KMP doses (Dabeek & Marra, 2019; Pietta, 2000; Terao, 2009), whereas preclinical studies have not demonstrated this effect after oral administration (Nirmala & Ramanathan, 2011). Considering these conflicting findings, further research on the safety of KMP intake is required. Clinical studies using KMP aglycone or KMP aglycone‐rich foods are essential for verifying their safety and impact on human health and are necessary to promote the effective use of KMP in humans. Therefore, in this study, KMP aglycone‐containing supplement was developed by employing an enzymatic treatment of horseradish leaves (Ikeda et al., 2020), which contain high KMP levels (Mizokami et al., 2021). Given that previous preclinical toxicity studies have confirmed that KMP aglycones are safe (Kimoto et al., 2022), a clinical trial was conducted using this in‐house‐developed product to investigate the safety of a high intake of KMP aglycones.

Participants received supplements containing 50‐mg KMP aglycone or placebo for 4 consecutive weeks. Significant differences were observed between the placebo and KMP groups for MCHC, direct bilirubin, γ‐GTP, AST, ALT, Na, P, and urine pH values (Tables 3, 4, 5). However, all parameters that varied between groups were within the reference values and were confirmed as not abnormal by a physician. Thus, although statistical differences were observed, they were not considered clinically significant. Other parameters in hematological, biochemical, or urinary analysis and participant characteristics values did not change significantly between groups (Tables 2, 3, 4, 5, 6). Additionally, no changes in laboratory values were observed for the age groups (20–39, 40–59, and 60–79 years) compared to those in the placebo group (data not shown). Furthermore, no adverse events from supplementation were observed (Table 7), indicating that continuous intake of 50‐mg KMP aglycone for 4 weeks is safe. No abnormal changes were observed in renal function markers such as creatinine and BUN or liver function markers such as γ‐GTP, AST, and ALT in the KMP aglycone group compared to those in the placebo group (Table 4), suggesting that aglycon had no harmful effect on the kidney and liver in these circumstances.

KMP intake has been shown to not affect indices of general toxicity in a 28‐day subacute study in mice (Yangzom et al., 2022) and a 13‐week subchronic study in rats (Kimoto et al., 2022); this study also confirmed similar results, for the first time, in a clinical trial in healthy adults receiving a high dose of KMP aglycone. Furthermore, much safety information has been reported for quercetin, a KMP analog (Andres et al., 2018). Quercetin is present in foods as glycosides but is often consumed as aglycone in dietary supplements (Andres et al., 2018). Estimated daily quercetin intakes range between 3 and 40 mg; however, higher doses have been shown to be effective and safe. A continuous intake of 150‐mg quercetin aglycone for 6 weeks, a quantity approximately 3–50 times higher than the estimated daily intake, had no effect on body weight or hepatic or renal function (Egert et al., 2009). No adverse events have been reported at higher doses of 500 mg/day, administered for 4–8 weeks (Javadi et al., 2017; Shi & Williamson, 2016). Given these facts, it is conceivable that ingesting KMP, even at higher doses, may be safe; however, more safety information is required.

This study had several limitations. It had a small sample size, which may lead to different results when tested at the population level. Furthermore, this study was not conducted under strict dietary restrictions; thus, it included the effects of individual dietary habits, and it could not be concluded that KMP intake is safe for all humans, given that this clinical trial was conducted on healthy adults. For example, the safety of KMP intake should be carefully investigated in people with underlying diseases, children, and pregnant women, considering that flavonoids readily cross the placenta (Skibola & Smith, 2000). Additionally, given that KMP intake may reduce iron bioavailability and/or the cellular level of folic acid (Devi et al., 2015), a safety evaluation of aglycon use in patients with iron and/or folic acid deficiency may also be required. Interactions between KMP and various food combinations should also be studied, as the absorption of KMP is approximately 2‐fold higher when KMP is consumed simultaneously with rutin (quercetin‐3‐*O*‐rutinoside) (Hashimoto et al., 2006), which is found in buckwheat or caper berries (Francesca et al., 2016; Suzuki et al., 2021).

KMP increases ATP content in vitro and in vivo (Akiyama et al., 2022); thus, increasing tissue ATP content by ingesting KMP may lead to improved exercise performance and reduced age‐related functional decline. KMP administration to a rat model of cerebral palsy was recently reported to attenuate gait deficits and impaired muscle strength (Visco et al., 2023). Although the effect of ATP on these effects was not examined in the report, the increase in ATP content due to KMP administration may have prevented muscle strength deficits. Clinical trials should be conducted to elucidate the possible beneficial effect of KMP intake in humans. The results of this study are the first to demonstrate the safety of high doses of KMP aglycone and offer basic safety information essential for designing more clinical trials to investigate its beneficial and therapeutical effects and assess its safety in special categories of patients.

## AUTHOR CONTRIBUTIONS

**Minoru Akiyama:** Conceptualization (equal); data curation (equal); formal analysis (equal); investigation (equal); methodology (equal); visualization (equal); writing – original draft (lead). **Tsubasa Mizokami:** Data curation (equal); formal analysis (equal); investigation (equal); visualization (equal). **Hikaru Ito:** Formal analysis (equal); investigation (equal). **Yasutaka Ikeda:** Conceptualization (equal); data curation (equal); methodology (equal); writing – review and editing (lead).

## FUNDING INFORMATION

This research received no specific grant from funding agencies in the public, commercial, or not‐for‐profit sectors.

## CONFLICT OF INTEREST STATEMENT

All authors are employees of Otsuka Pharmaceutical Co., Ltd.

## ETHICS STATEMENT

All study procedures were approved by the ethics committee of AMC Nishi‐Umeda Clinic and were conducted in accordance with the standards of the 1964 Declaration of Helsinki and its later amendments.

## INFORMED CONSENT

Informed consent was obtained from all participants included in the study.

## Data Availability

The data that support the findings of this study are available on request from the corresponding author. The data are not publicly available due to privacy or ethical restrictions.
